# Receptor for Advanced Glycation End Products - Membrane Type1 Matrix Metalloproteinase Axis Regulates Tissue Factor Expression via RhoA and Rac1 Activation in High-Mobility Group Box-1 Stimulated Endothelial Cells

**DOI:** 10.1371/journal.pone.0114429

**Published:** 2014-12-09

**Authors:** Koichi Sugimoto, Hiroshi Ohkawara, Yuichi Nakamura, Yoh Takuwa, Toshiyuki Ishibashi, Yasuchika Takeishi

**Affiliations:** 1 Department of Cardiology and Hematology, Fukushima Medical University, Fukushima, Japan; 2 Department of Physiology, Kanazawa University Graduate School of Medicine, Kanazawa, Japan; 3 Internal Medicine, Ohara General Hospital, Fukushima, Japan; Osaka University Graduate School of Medicine, Japan

## Abstract

**Background:**

Atherosclerosis is understood to be a blood vessel inflammation. High-mobility group box-1 (HMGB-1) plays a key role in the systemic inflammation. Tissue factor (TF) is known to lead to inflammation which promotes thrombus formation. Membrane type1 matrix metalloprotease (MT1-MMP) associates with advanced glycation endproducts (AGE) triggered-TF protein expression and phosphorylation of NF-κB. However, it is still unclear about the correlation of MT1-MMP and HMBG-1-mediated TF expression. In this study, we investigated the molecular mechanisms of TF expression in response to HMGB-1 stimulation and the involvement of MT1-MMP in endothelial cells.

**Methods and Results:**

Pull-down assays and Western blotting revealed that HMGB-1 induced RhoA/Rac1 activation and NF-kB phosphorylation in cultured human aortic endothelial cells. HMGB-1 increased the activity of MT1-MMP, and inhibition of RAGE or MT1-MMP by siRNA suppressed HMGB-1-induced TF upregulation as well as HMGB-1-triggered RhoA/Rac1 activation and NF-kB phosphorylation.

**Conclusions:**

The present study showed that RAGE/MT1-MMP axis modified HMBG-1-mediated TF expression through RhoA and Rac1 activation and NF-κB phosphorylation in endothelial cells. These results suggested that MT1-MMP was involved in vascular inflammation and might be a good target for treating atherosclerosis.

## Introduction

Currently, the pathogenesis of atherosclerosis is understood as vascular inflammation [1]. Endothelial dysfunction is an underlying condition that induces the inflammatory response. The imbalance of production of endothelium-derived bioactive substances induces release of new biologically active substances such as tumor necrotizing factor (TNF)-α and interleukins from macrophages. As a result, vascular smooth muscle cells cause differentiation, proliferation and migration. Apoptosis of macrophage brings T lymphocyte mobilization and progress of proliferative lesions.

Decreased production of nitric oxide (NO) and increased generation of reactive oxygen species (ROS) are known to further exacerbate endothelial cell dysfunction. Activation of small GTP-binding proteins RhoA destabilizes eNOS mRNA [2]. Rac1 is a source of constituent molecules of NADPH oxidase which produce ROS in the vessel wall [3]. Therefore, the role of small GTP-binding proteins in endothelial dysfunction has been noted.

Lysophosphatidylcholine (LPC) is a phospholipid component of oxidized LDL. We found that LPC induced rapid RhoA activation [4] and actin stress fiber reorganization lead to destabilization of eNOSmRNA in endothelial cells [5]. In addition, membrane type1 matrix metalloproteinase (MT1-MMP) has been shown to function as a signaling molecule in vascular remodeling [6], [7]. Previously we reported that signaling pathways of RhoA/Rac1 activation induced by oxidized LDL are mediated via lectin-like oxidized LDL receptor-1 (LOX-1) and proved that for the first time MT1-MMP is upstream of RhoA/Rac1 activation. In addition, we clarified that MT1-MMP forms a complex with LOX-1 and that modifies its function [8]. These findings suggest that MT1-MMP is also deeply involved in inflammation via endothelial dysfunction.

High-mobility group box-1 (HMGB-1) is nuclear DNA binding proteins which are released from stimulated monocytes, inflammatory macrophages and necrotizing cells. It is known to be a very important mediator in systemic inflammatory diseases such as sepsis and endotoxemia [9], [10].

Receptor for advanced glycation end products (RAGE) has been reported as one of the receptors for HMGB-1. Transcription factor NF-κB is activated by binding of ligands to RAGE including HMGB-1 and leads to the production of various inflammatory cytokines and tissue factor (TF) synthesis [11], [12].

Interestingly, we found that activation of Rac/NADPH oxidase and phosphorylation of NF-κB mediated via RAGE were suppressed by MT1-MMP inhibition, which decreased production of TF, monocyte chemotactic protein-1 (MCP-1) in rabbit smooth muscle cells [13], [14]. These results suggested the involvement of MT1-MMP in the cellular signaling system of HMGB-1/RAGE mediated via NF-κB. However, there is no report on the association of MMP with HMGB-1/RAGE.

Therefore, in the present study, we aimed to clarify the role of HMGB-1 and RAGE-MT1-MMP axis on signal transduction and the development of atherosclerosis.

## Methods

### Materials

The sources of most of the conventional reagents has been described previously [15], [16]. We obtained human recombinant HMGB-1 from ATGen Co., Ltd (Seongnam, South Korea).

### Preparation of Endothelial Cells

Human aortic endothelial cells (HAECs) were cultured according to the suppliers' instructions (Clonetics Inc., Walkersville, MD and Sanko Junyaku Co., Ltd., Tokyo, Japan) and used for all experiments after 5 to 10 passages [4], [17].

### Western Blotting

We determined the expression of RhoA, Rac1, TF and α-tubulin by Western blotting [4], [17], [18]. We used mouse monoclonal antibodies to RhoA (Santa Cruz Biotechnology, Santa Cruz, CA) and Rac1 (Upstate Biotechnology, Lake Placid, NY) and α-tubulin (Santa Cruz Biotechnology) at a dilution of 1∶500, and to TF (Transduction Laboratories, Lexington, KY) diluted 1∶1000 for immunoblotting. The signals from immunoreactive bands were visualized by an Amersham ECL system (Amersham Pharmacia Biotech UK Ltd., Buckinghamshire, England).

### GTP/GDP Exchange of RhoA and Rac1

GTP-bound active forms of RhoA and Rac1 were determined by pull-down assay as described previously [4], [17], [18]. Extracts of HAECs were incubated at 4°C for 45 min with glutathione-Sepharose 4B beads coupled with glutathione-S-transferase (GST)-rhotekin fusion protein for determination of RhoA activity or GST-p21-activated kinase (PAK) for determination of Rac1 activity. Bound RhoA and Rac1 were semi-quantitatively detected by Western blotting.

### Determination of NF-κB Activation

NF-κB phosphorylation was determined by Western blotting using rabbit polyclonal antibodies against phosphorylated and unphosphorylated NF-κB p65 (Ser536, dilution 1∶1000; Cell Signaling Technology Inc., Beverly, MA), as described previously [18].

### Measurement of MT1-MMP Activity of Membrane Fractions

As described previously, [4], [8], [18] we prepared membrane fractions of HAECs to evaluate the activity of MT1-MMP. Briefly, cells were lysed with a hypotonic buffer, then sonicated and centrifuged at 15,000 g for 10 minutes. The separated membrane fractions were assessed by the commercially available fluorescent assay kit (SensoLyte 520 MMP-14 assay kit, AnaSpec, San Jose, CA) according to the manufacturer's instructions.

### siRNA

MT1-MMP and RAGE expression were silenced by transfection of small interfering RNA (siRNA) 5′-CUGGCAGUUCGGCUAGAUUUC-3′ (sense strand for MT1-MMP) and 5′-CACUGCAGUCGGAGCUAAUGG-3′ (sense strand for RAGE) (RNAi Co., Ltd., Tokyo, Japan) [19]. HAECs were transfected with double-strand siRNA in serum-free medium mixed with oligofectamine (Invitrogen, Carlsbad, CA) according to the manufacturer's instructions. Four hours after transfection, HAECs were incubated in a medium containing 2% fetal bovine serum (FBS) for 48 h. Alternatively, cells were treated with an irrelevant siRNA 5′-GUACCGCACGUCAUUCGCAUC-3′ (sense strand) as a negative control.

### Fluorescent immunostaining

HAECs cultured on chamber slides were fixed in 10% formalin for 10 min, and then incubated with a rabbit polyclonal antibody to RAGE (Santa Cruz Biotechnology) and a mouse monoclonal antibody to MT1-MMP (DAIICHI Fine Chemical Co., Ltd., Toyama, Japan) at room temperature for 60 min. After washing, anti-mouse Alexa 488 and anti-rabbit Alexa 594 (Molecular Probes, Eugene, OR, USA) were reacted for 60 min. Stained cells were stored in the dark until they were analyzed by a fluorescence microscope (Olympus, Tokyo, Japan).

### Immunoprecipitation

HAECs were extracted in a RIPA buffer (Sigma–Aldrich), and the lysates were centrifuged at 10000 g at 4°C. The supernatant was precleared and reacted with anti-MT1-MMP antibody (DAIICHI Fine Chemical) at a concentration of 1.0 mg/mL, respectively. Immunoprecipitated protein was resolved by sodium dodecyl sulfate–polyacrylamide gel electrophoresis, followed by western blotting of RAGE, respectively. We used the primary antibody against RAGE (Santa Cruz) and anti-rabbit IgG True blot (eBioscience, Inc., San Diego, CA, USA), which detects native antibody but not the denatured 55 kDa heavy chain and 23 kDa light chains of the immunoprecipitating antibody, as the secondary antibody.

### Densitometric Analysis and Statistical Analyses

After scanning blots into a computer (EPSON GT5500ART, Tokyo, Japan), the optical densities of individual immunoblots were analyzed using the NIH IMAGE Program software as described previously [8], [16]. Statistical analyses were performed using ANOVA with Scheffé's post hoc test if appropriate. A value of *P*<0.05 was considered significant.

## Results

### Effect of HMGB-1 RhoA, Rac1 Activation in Endothelial Cells

**Figure 1:**
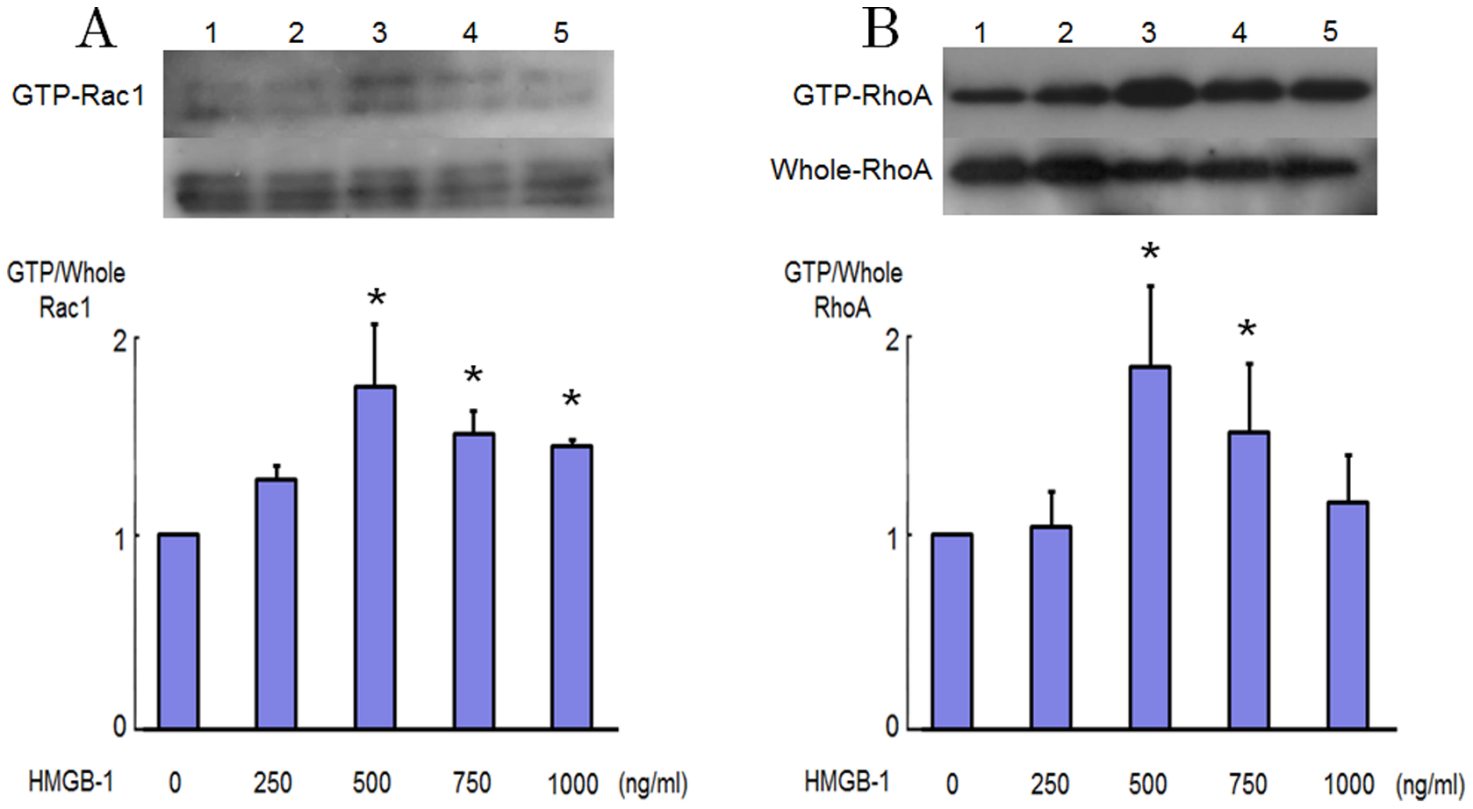
RhoA and Rac1 activation stimulated by HMGB-1 in endothelial cells. Levels of GTP-bound active forms of RhoA and Rac1 as determined by pull-down assays in cultured human aortic endothelial cells (HAECs) stimulated by 250 to 1000 ng/ml HMGB-1. Bars are mean±S.D. of quantitative densitometric analyses from 3 separate experiments. Representative immunoblots from 3 separate experiments are shown. ^*^ *P*<0.05 vs. Lane 1.

**Figure 2:**
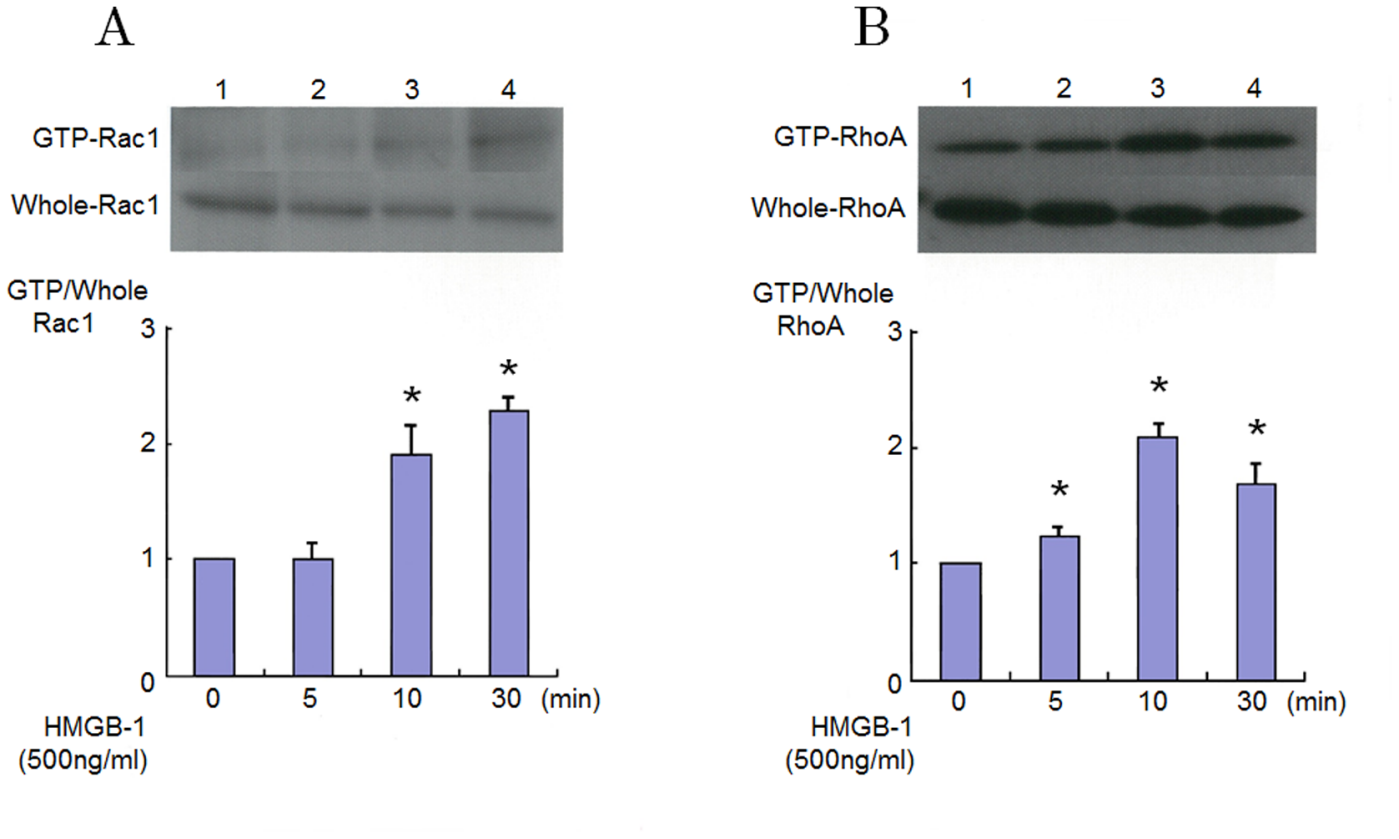
Rapid RhoA and Rac1 activation in HMGB-1 stimulated endothelial cells. Levels of GTP-bound active forms of RhoA and Rac1 as determined by pull-down assays in cultured HAECs 5 to 30 minutes after adding 500 ng/ml HMGB-1. Bars are mean±S.D. of quantitative densitometric analyses from 4 separate experiments. Representative immunoblots from 4 separate experiments are shown. ^*^ *P*<0.05 vs. Lane 1.

Signaling effect of HMGB-1 on endothelial dysfunction is still largely unknown. We therefore investigated the effect of HMGB-1 on RhoA and Rac1 activation in HAECs. Fig. 1A and 1B show the RhoA and Rac1 activation in response to HMGB-1. We confirmed that activation of RhoA and Rac1 is recognized when the concentration of HMGB-1 become more than 500 ng/ml (Fig. 1). As shown in Fig. 2A and 2B, pull-down assays demonstrated that stimulation by HMGB-1 increased the level of the GTP-bound forms of RhoA and Rac1, whereas the levels of RhoA and Rac1 in whole cell lysates were not changed by HMGB-1 in HAECs. The GTP-loading of RhoA was induced within 5 min and peaked after 10 min adding HMGB-1. The GTP-loading of Rac1 appeared slightly later than that of RhoA and persisted for at least up to 30 min after HMGB-1 stimulation.

### Role of RAGE on RhoA and Rac1 Activation Induced by HMGB-1

**Figure 3:**
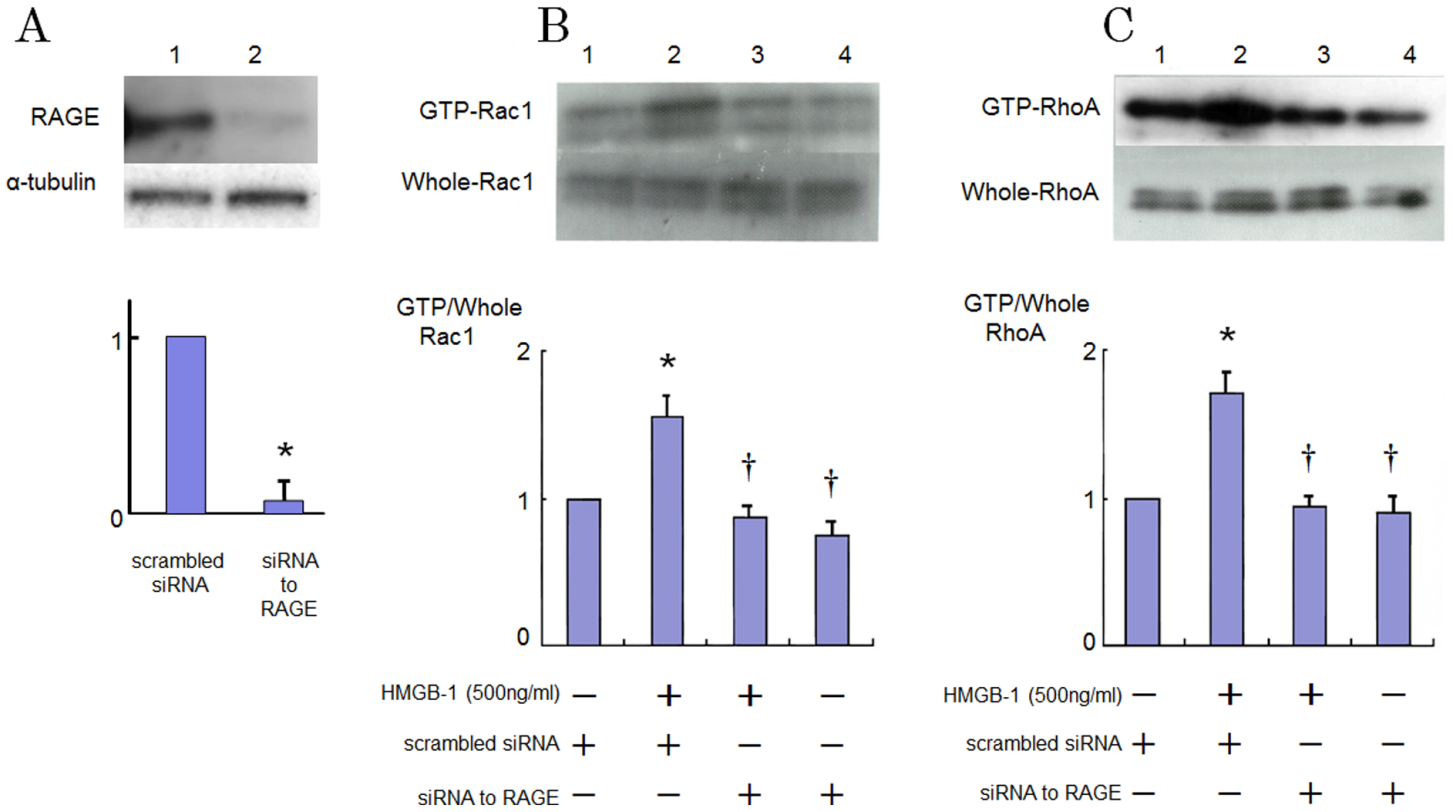
Effects of inhibition of RAGE with siRNA on increased GTP-loading of RhoA and Rac1 caused by HMGB-1 in HAECs. (A) Effect of knockdown of RAGE by siRNA on the RAGE protein level as determined by western blotting. Bars are mean ±S.D. of quantitative densitometric analyses from 3 separate experiments. Representative immunoblots from 3 independent experiments are shown. *P<0.05 vs. Lane 1. (B and C) Cells were incubated in the presence of HMGB-1 with or without transfection with siRNA to RAGE. Bars are mean±S.D. of 4 separate experiments. Representative immunoblots from 4 separate experiments are shown. ^*^ *P*<0.05 vs Lane 1; ^†^ *P*<0.05 vs. Lane 2.

RAGE is one of the endothelial receptors for HMGB-1 [11]. We examined the effect of inhibition of RAGE on the HMGB-1-induced RhoA and Rac1 activation. Fig. 3A shows that a siRNA-mediated knockdown of RAGE reduced RAGE protein levels by approximately 90% as compared to the scrambled negative control. Induction of siRNA for RAGE abrogated the GTP-loading of RhoA and Rac1 5 and 15 min after exposure to HMGB-1, respectively (Fig. 3B and 3C).

### MT1-MMP Activity

**Figure 4:**
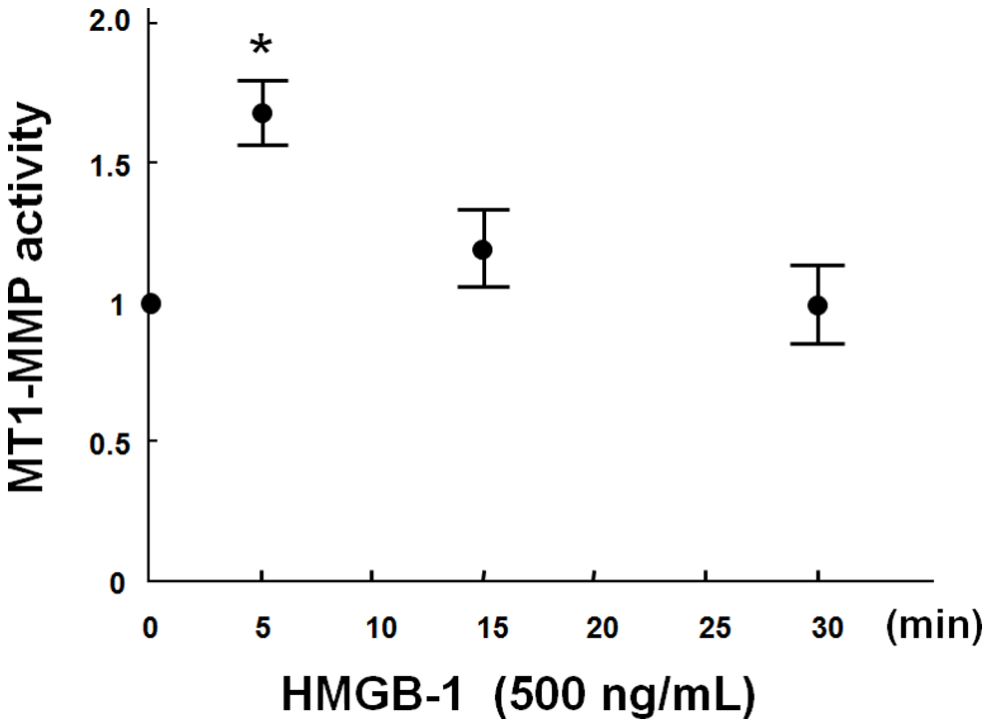
HMGB-1 increases MT1-MMP activity in cultured endothelial cells. Plasma membrane fractions were extracted from HAECs. Changes in the activity of MT1-MMP are shown after HMGB-1 stimulation. Results are expressed as mean±S.D. of 3 separate experiments performed in pentaplicate. ^*^ *P*<0.05 vs. 0 minute before HMGB-1 stimulation.

Previously, we have reported that substances to elicit arteriosclerosis such as oxidized LDL raise the MT1-MMP activity of vascular endothelial cells [8]. Therefore, we measured the activity of MT1-MMP in HMGB-1-stimulated HAECs. We found a significant increase of MT1-MMP activity in HAECs as a result of addition of HMGB1 at a concentration of 500 ng/ml in the culture supernatant. The MT1-MMP activation induced by HMGB-1 was observed 5 minutes after HMGB-1 addition and returned to control after 30 minutes of HMGB-1 stimulation (Fig. 4).

### Role of MT1-MMP on HMGB-1-Induced RhoA and Rac1 Activation

**Figure 5:**
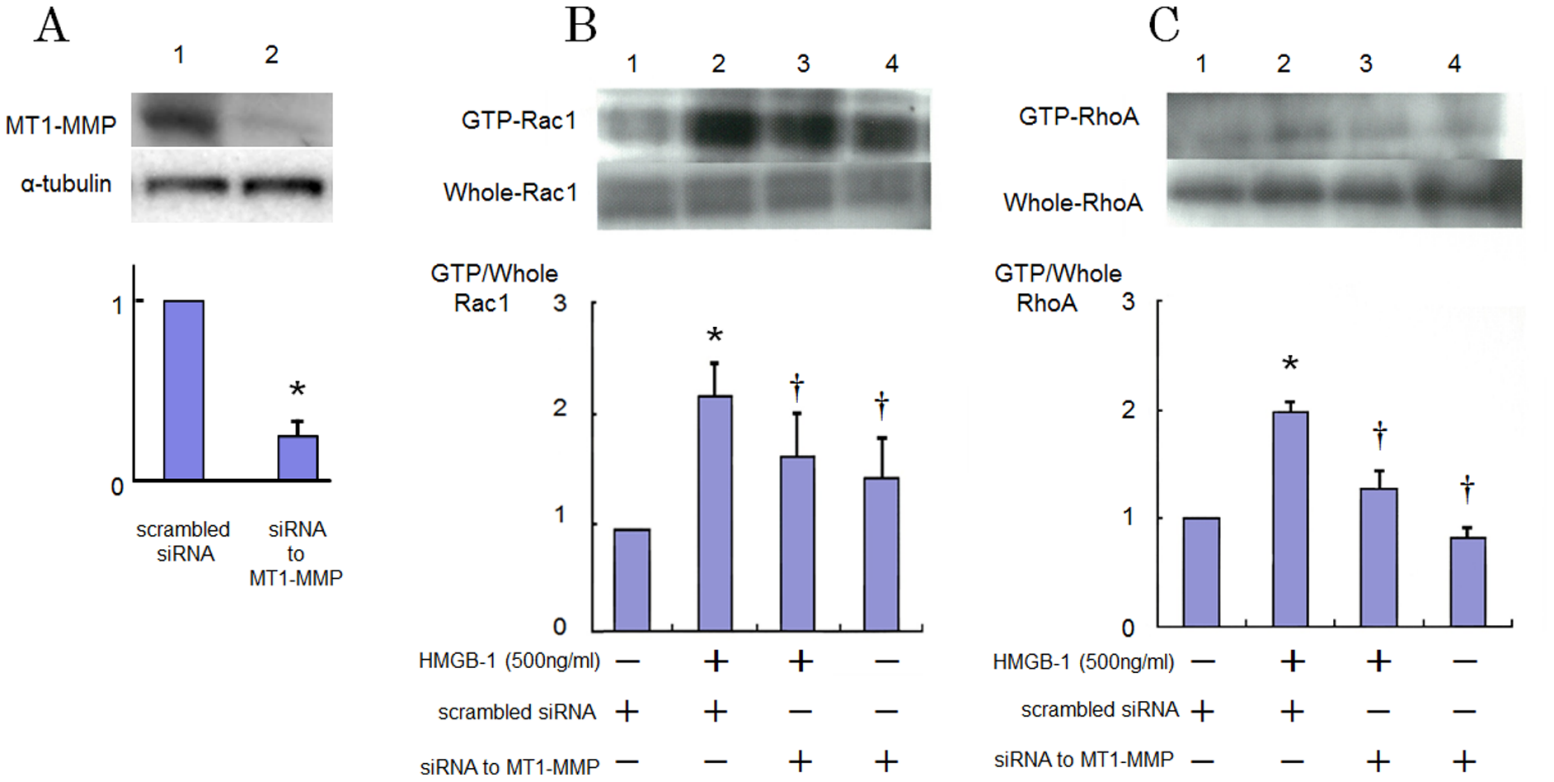
Effects of inhibition of MT1-MMP with siRNA on increased GTP-loading of RhoA and Rac1 caused by HMGB-1 in HAECs. (A) Effect of knockdown of MT1-MMP by siRNA on the MT1-MMP protein level as determined by western blotting. Bars are mean ±S.D. of quantitative densitometric analyses from 3 separate experiments. Representative immunoblots from 3 independent experiments are shown. *P<0.05 vs. Lane 1. (B and C) Cells were incubated in the presence of HMGB-1 with or without transfection with siRNA to MT1-MMP. Bars are mean±S.D. of 4 separate experiments. Representative immunoblots from 4 independent experiments are shown. ^*^ *P*<0.05 vs Lane 1; ^†^ *P*<0.05 vs. Lane 2.

Then we conducted experiments on the role of MT1-MMP on the HMGB-1-induced signaling pathway in HAECs. Prior to stimulation with HMGB-1, HAECs were transfected to siRNA for MT1-MMP. Expression of MT1-MMP protein is inhibited about 70% by siRNA (Fig. 5A). As shown in Fig. 5B and 5C, the activation of Rac1 and RhoA induced by HMGB-1 was suppressed in the MT1-MMP-silenced cells.

### Association of RAGE and MT1-MMP

**Figure 6:**
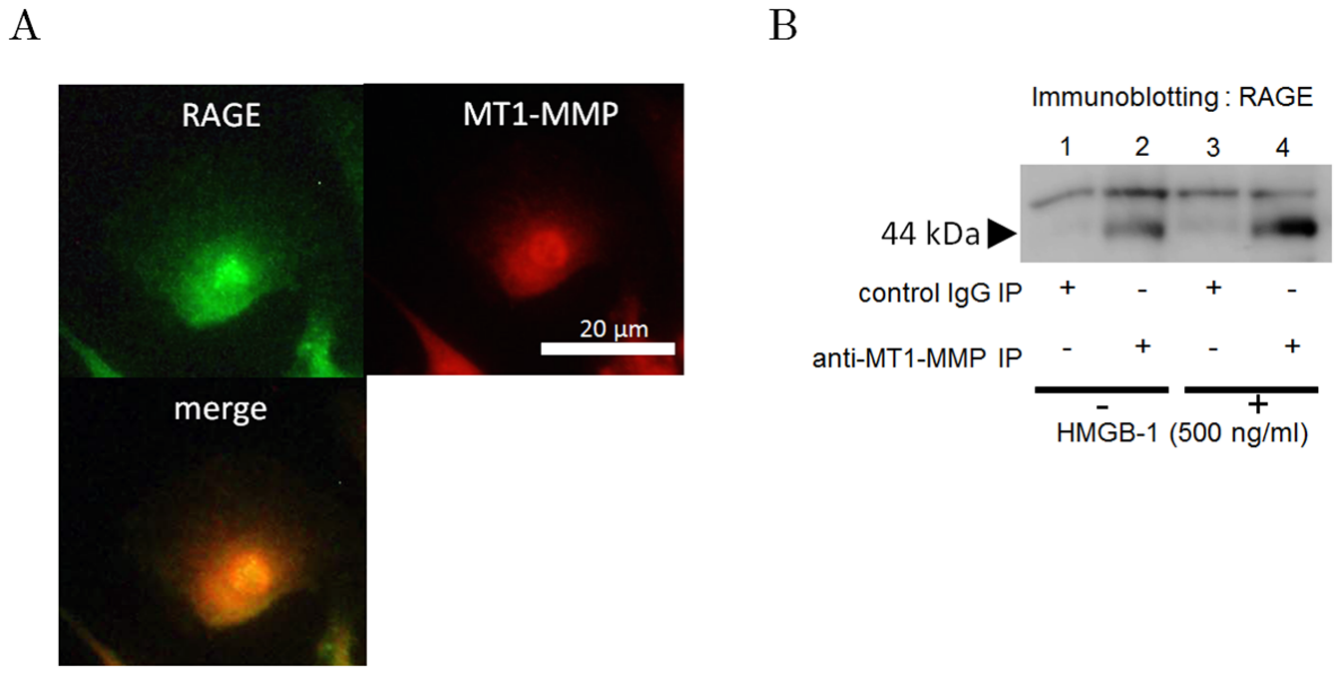
Colocalization and binding of RAGE and MT1-MMP in endothelial cells. (A) Association of RAGE and MT1-MMP according to fluorescent immunohistochemistry. Merged image indicates that RAGE is partially colocalized with MT1-MMP. Photomicrographs are from an experiment representative of 3 independent experiments. (B) Formation of a complex of RAGE and MT1-MMP as determined by immunoprecipitation with or without HMGB-1 stimulation. Immunoprecipitates made using an isotype-matched control antibody did not show 44-kDa band (Fig. 6B, lane 1 and 3), whereas 44 kDa band recognized by immunoblotting with anti-RAGE antibody was detected in the MT1-MMP-immunoprecipitates (Fig. 6B, lane 2 and 4).

The immunostaining was performed to observe the distributions of MT1-MMP and RAGE in HAECs. Fig. 6A shows that MT1-MMP partially localized with RAGE.

Further, the immunoprecipitation was carried out to reveal the interaction of MT1-MMP and RAGE. The band of 44 kDa of RAGE was detected in the immunoprecipitates with the primary antibody to MT1-MMP. In contrast, in the immunoprecipitates with negative control IgG, only a faint band was recognized (Fig. 6B). These suggested that MT1-MMP forms a complex with RAGE. The binding of MT1-MMP and RAGE was found in both non-stimulated cells and HMGB-1-stimulated cells. Association of RAGE and MT1-MMP was not different between basal condition and stimulation with HMGB-1.

### Role of RAGE-MT1-MMP Axis in HMGB-1-Induced NF-κB Phosphorylation and TF Protein Expression

Previously, we have shown that inhibition of RhoA suppressed phosphorylation of NF-kB in HAECs [18] and silencing of MT1-MMP suppressed the AGE triggered-TF protein expression and phsphorylation of NF-κB in smooth muscle cells [14].

These facts suggest that expression of TF antigen is mediated via RAGE and NF-κB in HMGB-1-stimulated HAECs, and that MT1-MMP plays an important role in this signaling.

**Figure 7:**
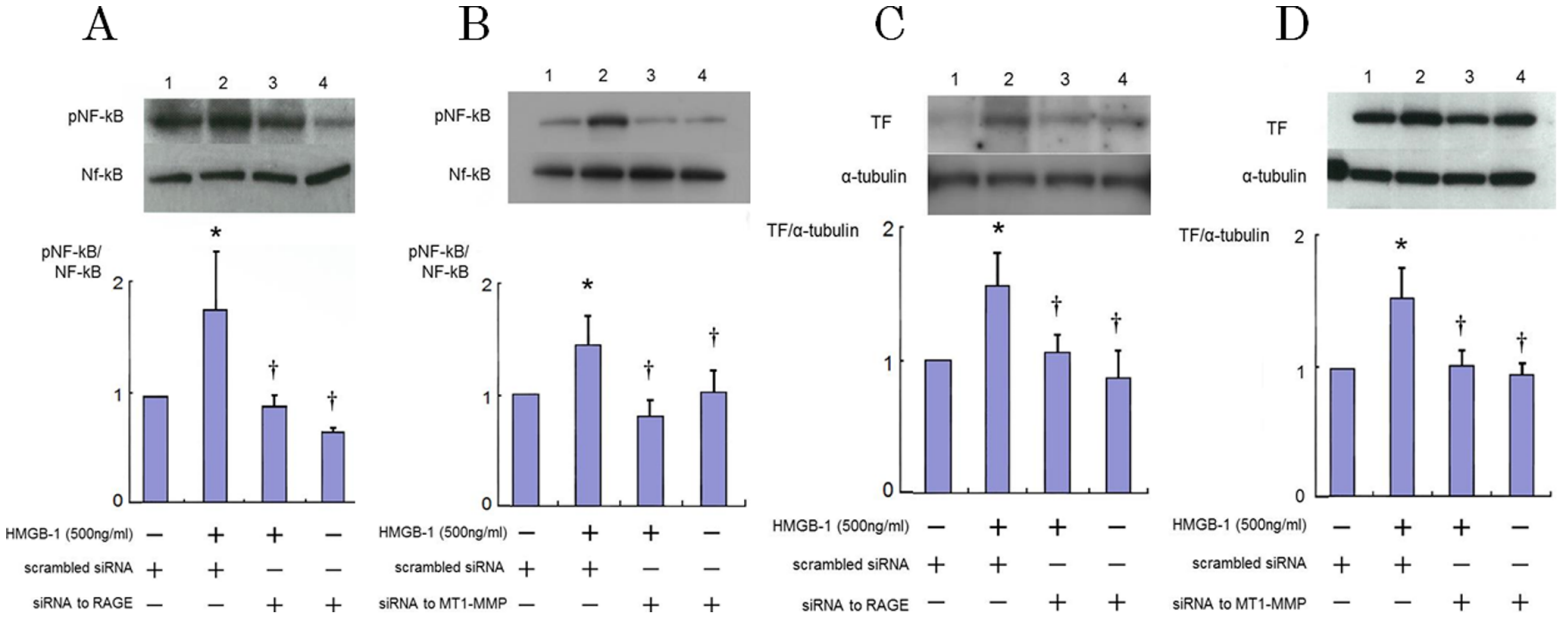
Role of RAGE and MT1-MMP in NF-κB phosphorylation (A, B) and HMGB-1-triggered upregulation of TF (C, D) in endothelial cells. HAECs were transfected with siRNA to RAGE or MT1-MMP and then stimulated by HMGB-1 for 30min (A, B) or 18 hours (C, D), followed by Western blotting. Immunoblots are from an experiment representative of 3 similar experiments. Quantitative results of TF (obtained by densitometry) were normalized for α-tubulin levels. Bars are mean±S.D. of quantitative densitometric analyses from 3 separate experiments. ^*^ *P*<0.05 vs Lane 1; ^†^ *P*<0.05 vs. Lane 2.

Therefore, we examined the role of MT1-MMP and RAGE in NF-κB phosphorylation and TF expression in HAECs in response to HMGB-1. We revealed that HMGB-1 increased the levels of NF-κB p65 phosphorylation 1 hour after their addition, although the levels of nonphosphorylated NF-κB p65 were not changed by HMGB-1. Silencing of RAGE and MT1-MMP prevented the activation of NF-κB p65 induced by HMGB-1 in HAECs (Fig. 7A and 7B). Theses indicated an integral role of RAGE-MT1-MMP axis in HMGB-1-induced NF-κB phosphorylation. As shown in Fig. 7C and 7D, TF protein expression was enhanced by HMGB-1 stimulation in HAECs. Inhibition of RAGE by siRNA suppressed the HMGB-1-induced TF expression in HAECs (Fig. 7C). These indicated that HMGB-1-triggered TF expression mediated via RAGE in HAECs. In addition, transfection of siRNA for MT1-MMP significantly reversed the HMGB-1-induced upregulation of TF protein expression in HAECs (Fig. 7D). These findings suggested that MT1-MMP is involved in the HMGB-1-induced TF expression via RAGE in HAECs.

## Discussion

In this study, we have shown that binding of RAGE and HMGB-1 induced RhoA and Rac1 activation, phosphorylation of NF-κB leading to upregulation of TF protein expression in HAECs. We also demonstrated that HMGB-1 increased the MT1-MMP activity which modified the HMGB-1 signaling.

According to previous reports including our own, MT1-MMP is known to form a complex on the cell surface with various receptors such as CD44, PDGF receptor, and LOX-1 [6], [7], [8]. Furthermore, we have demonstrated not only the binding of MT1-MMP to RAGE but also the colocalization of these molecules using immunostaining and immunoprecipitation in smooth muscle cells [14]. In this study, we clarified for the first time that the binding of MT1-MMP to RAGE was also occurring in human vascular endothelial cells (Fig. 6). However, more detailed mechanisms such as binding site or how to modulate are still unclear.

Though it is widely known that the expression and activity of MT1-MMP are increased by various inflammatory mediators, [20] this is, to our knowledge, the first report to show the activation of MT1-MMP by HMGB-1.

In the results of our study, the activation of MT1-MMP has been observed in a few minutes in addition to HMGB-1 to HAECs which was an early reaction rather than a de novo synthesis of MT1-MMP protein enhanced by HMGB-1. The activity of MT1-MMP induced by HMGB-1 returned to the control level in 30 minutes, whereas the activation of MT1-MMP by oxidization LDL continued for about 30 minutes [8]. The mechanism of this difference remains unclear.

The presence of Src-dependent phosphorylation of MT1-MMP in bovine endothelial cells has been reported by Nyalendo et al [21]. We cannot rule out the possibility that phosphorylation of MT1-MMP may be involved in the rapid MT1-MMP activation caused by HMGB-1 stimulation. In addition, the activity of MT1-MMP that we have measured was the activity as a proteolytic enzyme of MT1-MMP. There may be a need to consider this separately from the activation of a signaling molecule.

Downregulation of MT1-MMP by siRNA prevented the GTP/GDP exchange of small GTP proteins. This indicated that MT1-MMP not only combined with the receptor, but acted functionally. This effect was similarly observed even with endogenous inhibitors such as tissue inhibitor of metalloprotease-2 as well as downregulation of MT1-MMP by siRNA [8].

On the other hand, the suppression of the phosphorylation of NF-κB did not seem to be the direct effect of the MT1-MMP inhibition but rather a result of the RhoA inhibition in the MT1-MMP-silenced cells, since we recognized that RhoA inhibition by C3 exoenzyme, a chemical RhoA inhibitor, was suppressed phosphorylation of NF-κB by monocyte adhesion, which suggested RhoA was upstream of NF-κB [18]. Nakakuki et al. also reported that relationship between RhoA and NF-κB. They showed that Y-27632, a chemical inhibitor of Rho-kinase, blocked CRP-induced NF-κB reporter gene expression [22]. These results suggest that RhoA is upstream of NF-κB in endothelial cells. It is known that expression of TF is controlled by NF-κB [12]. Taken together, our results clarified that MT1-MMP suppressed HMGB-1-induced TF upregulation via RAGE, RhoA and NF-κB signaling in HAECs.

Previously, we reported that Rac1 inhibition suppressed TF expression mediated via reactive oxygen species (ROS) generation in thrombin-stimulated endothelial cells [23]. We suppose that HMGB-1-stimulated Rac1 activation also induces NADPH oxidase-mediated ROS generation and regulates TF expression which is one of the redox-sensitive signaling-dependent molecule.

It is known that the Toll like receptor (TLR)-2 and TLR-4 expressed in vascular endothelial cells and function as the receptor for HMGB-1 [24]. Thus, it is expected that TLR-2 or TLR-4-mediated NF-κB phosphorylation regulates TF expression. Further experiment with knockdown or blocking TLRs is necessary to confirm the involvement of TLRs in HMGB-1-induced TF expression in future study.

## Conclusions

Our present data suggest that HMGB-1 activates MT1-MMP through RAGE lead ing to RhoA/Rac1 activation, NF-κB phosphorylation and TF protein upregulation as a pathology of the progression of atherosclerosis and vascular endothelial dysfunction.

Our viewpoint that MT1-MMP takes part in the mechanisms of the inflammation in atherosclerosis is unique. The results of this study will bring about new developments in the strategy for the treatment of arteriosclerosis as vascular inflammation.
